# Estimation of renal perfusion based on measurement of rubidium-82 clearance by PET/CT scanning in healthy subjects

**DOI:** 10.1186/s40658-021-00389-0

**Published:** 2021-05-31

**Authors:** Stine Sundgaard Langaa, Thomas Guldager Lauridsen, Frank Holden Mose, Claire Anne Fynbo, Jørn Theil, Jesper Nørgaard Bech

**Affiliations:** 1 Gødstrup HospitalUniversity Clinic in Nephrology and Hypertension, Department of Medical Research, Gødstrup Hospital and Aarhus University, Lægaardvej 12J, 7500 Holstebro, Denmark; 2 Department of Nuclear Medicine, Gødstrup Hospital, Herning, Denmark; 3 Department of Clinical Medicine, Aarhus University, Aarhus, Denmark

**Keywords:** PET/CT, Rubidium-82, Pharmacokinetic modelling, Renal blood flow, Effective renal plasma flow, Renal volumes

## Abstract

**Background:**

Changes in renal blood flow (RBF) may play a pathophysiological role in hypertension and kidney disease. However, RBF determination in humans has proven difficult. We aimed to confirm the feasibility of RBF estimation based on positron emission tomography/computed tomography (PET/CT) and rubidium-82 (^82^Rb) using the abdominal aorta as input function in a 1-tissue compartment model.

**Methods:**

Eighteen healthy subjects underwent two dynamic ^82^Rb PET/CT scans in two different fields of view (FOV). FOV-A included the left ventricular blood pool (LVBP), the abdominal aorta (AA) and the majority of the kidneys. FOV-B included AA and the kidneys in their entirety. In FOV-A, an input function was derived from LVBP and from AA, in FOV-B from AA. One-tissue compartmental modelling was performed using tissue time activity curves generated from volumes of interest (VOI) contouring the kidneys, where the renal clearance of ^82^Rb is represented by the K_1_ kinetic parameter. Total clearance for both kidneys was calculated by multiplying the K_1_ values with the volume of VOIs used for analysis. Intra-assay coefficients of variation and inter-observer variation were calculated.

**Results:**

For both kidneys, K_1_ values derived from AA did not differ significantly from values obtained from LVBP, neither were significant differences seen between AA in FOV-A and AA in FOV-B, nor between the right and left kidneys. For both kidneys, the intra-assay coefficients of variation were low (~ 5%) for both input functions. The measured K_1_ of 2.80 ml/min/cm^3^ translates to a total clearance for both kidneys of 766 ml/min/1.73 m^2^.

**Conclusion:**

Measurement of renal perfusion based on PET/CT and ^82^Rb using AA as input function in a 1-tissue compartment model is feasible in a single FOV. Based on previous studies showing ^82^Rb to be primarily present in plasma, the measured K_1_ clearance values are most likely representative of effective renal plasma flow (ERPF) rather than estimated RBF values, but as the accurate calculation of total clearance/flow is very much dependent on the analysed volume, a standardised definition for the employed renal volumes is needed to allow for proper comparison with standard ERPF and RBF reference methods.

**Supplementary Information:**

The online version contains supplementary material available at 10.1186/s40658-021-00389-0.

## Background

Kidney disease and hypertension are major contributors to the overall global disease burden. In the pathogenesis of acute kidney injury (AKI), renal ischemia, as a result of a reduction in total RBF, has been accepted as a significant factor. However, recent studies suggest that renal hypoperfusion may play a less important role [1, 2]. In fact, RBF measurements in sepsis-associated AKI have shown much discrepancy; reduced, normal or even increased RBF have been reported [3–5]. In patients with chronic kidney disease (CKD), RBF is reduced compared with controls [6, 7], possibly contributing to the progression of renal dysfunction. In renal circulation studies, most patients with essential hypertension display reduced RBF [8, 9], the greatest reduction demonstrated in malignant hypertension [9, 10]. Additionally, renal vasoconstriction has been identified in pre-hypertensive adults, indicating that renal vascular abnormalities could be a cause of hypertension rather than caused by hypertension [11, 12].

Quantification of renal perfusion in humans has proven difficult. Clearance-based methods estimating ERPF are time consuming and burdensome for patients [13–15], and alternative radiological imaging techniques assessing RBF, such as magnetic resonance imaging and ultrasonography, all have considerable limitations [7, 16]—none of which have been routinely implemented in clinical practise. Dynamic positron emission tomography (PET) using perfusion tracers is currently considered the most accurate, non-invasive method for determination of organ perfusion. Thus, with good homogeneity and high perfusion rate, the kidneys are well suited for PET studies.

PET scans using ^82^Rb are routinely performed to assess myocardial blood flow in patients suspected of ischemic heart disease [17, 18]. ^82^Rb is a potassium analogue with a short half-life of 75 s, produced in a generator by the radioactive decay of strontium-82 (^82^Sr). Due to its high first-pass renal extraction (~ 90%) and slow wash-out, ^82^Rb is well suited for mathematic modelling of RBF using dynamic PET-methods [19].

The first human ^82^Rb PET/CT study of renal perfusion showed high image quality, resolution and contrast, as well as demonstrated a high natural ^82^Rb renal uptake [20, 21]. RBF was evaluated using a 1-tissue compartment model, where the K_1_ parameter is presumed to represent estimated RBF.

Compartmental modelling requires an input function (IF) described by a blood-pool time-activity curve. In quantification of myocardial blood flow, the LVBP has been validated as an image-derived input function (IDIF) [22, 23], obviating the need for arterial blood sampling. However, the LVBP is not necessarily ideal for studying renal perfusion, as the LVBP and the kidneys in their entirety may not fit within a single limited axial scanner FOV. This is especially true for older PET scanners. In order, to ensure that an IF is estimated as correctly as possible, as well as to minimise radiation dose associated with the scanning, inclusion of the blood-pool and the kidneys in their entirety in the same FOV is important. This can be accomplished if the AA can replace the LVBP as IF in the model, as suggested by Tahari et al. [21].

To determine whether this method is suitable for clinical, reliable assessment of RBF, this study further investigates the substitution of AA as a valid alternative to LVBP by comparing the resulting K_1_ values obtained from use of the two different IFs in a substantially larger study population than that of [21]. We also evaluate method precision by determination of intra-assay coefficients of variation for both IFs as well as assess inter-observer variation. Furthermore, existing literature assumes that the perfusion quantity measured using ^82^Rb is estimated RBF [21, 24]. However, early investigations into the exchange rates of radioactive potassium and rubidium between plasma and erythrocytes showed rates of ~ 2% per hour [25, 26], implying that initially, and hence during renal uptake studies, the majority of injected ^82^Rb will be almost exclusively present in plasma. We discuss and question whether flow values measured by ^82^Rb clearance are actually representative of RBF or whether they should be interpreted as estimates of ERPF.

## Methods

### Study design

**Fig. 1:**
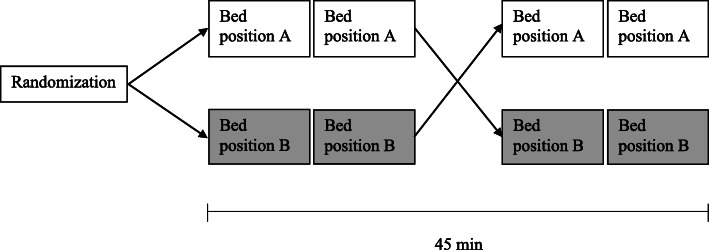
Study design

This study was performed as a randomised cross-over study (Fig. 1). During a period of approximately 45 min, each subject underwent four 8-min dynamic ^82^Rb PET/CT scans in two different bed positions, A and B (FOV-A and FOV-B). In each bed position, duplicate scans were performed.

### Participants

Healthy participants were recruited through advertisement, primarily at local educational institutions. Prior to inclusion, each participant completed a screening programme. Screening consisted of a medical history; a clinical examination including measurements of weight, height and blood pressure; electrocardiography; and blood tests to determine electrolytes, creatinine, albumin, alanine aminotransferase (ALAT), leucocytes, haemoglobin, haematocrit and thrombocytes. Urine was screened for leucocytes, glucose, nitrite, ketones and haemoglobin. Inclusion criteria were men and women aged 18–40 years with a body mass index (BMI) in the range 18.5–30.0 kg/m^2^. Exclusion criteria were medical treatment (except hormonal contraceptives); pregnancy or breastfeeding; smoking; substance abuse; alcohol consumption >14 units^1^ per week for men and >7 units per week for women; signs of clinically relevant kidney disease, heart disease, liver disease or endocrine disease in the history, clinical examination, or the para-clinical tests; hypertension; neoplastic disease; and blood donation within 1 month of the examination day. Withdrawal criteria were development of exclusion criteria or withdrawal of consent.

### Number of subjects

Seventeen subjects are required to detect a 0.40 ml/min/cm^3^ difference in RBF (standard deviation (SD) 0.38 ml/min/cm^3^) for a 5% significance level and power of 80%. To allow for dropout, 20 subjects were included.

### Pre-scan procedure

For 24 h preceding the acquisition of PET/CT scans, fluid intake was standardised to 35 ml/kg body weight of still water; subjects maintained a free diet and were instructed to avoid strenuous exercise. Subjects arrived at 8 a.m. at the Department of Nuclear Medicine, Herning Hospital, Regional Hospital West Jutland, Denmark, after an overnight fast. In female subjects, pregnancy was ruled out.

### Radiopharmaceutical

On each day of examination, the ^82^Sr/^82^Rb generator (Cardiogen-82; Bracco Diagnostics Inc., Monroe Township, NJ, USA) was quality checked according to approved guidelines (Bracco Diagnostics Inc.) including test for breakthrough of ^82^Sr/^85^Sr. The generator was calibrated to deliver a dose of 555 MBq (15 mCi) ^82^Rb for each injection, which was administered automatically using a pre-programmed pump and infusion system. The subjects received four doses in total.

### PET/CT scanning

**Fig. 2:**
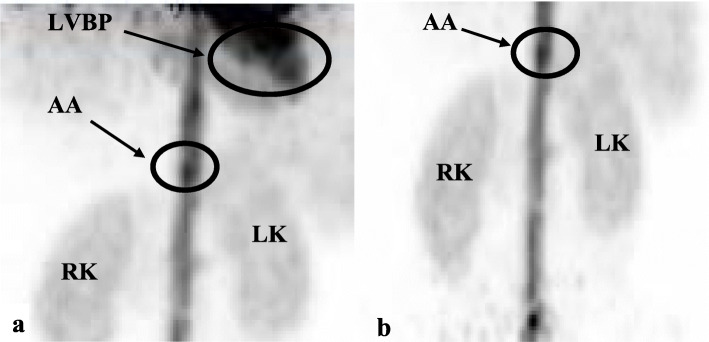
Anatomical contents within the 22-cm axial FOV for an example study image (subject 2): **a** FOV-A: bed position includes LVBP, AA and kidneys (body length and kidney size determine whether the kidneys can be seen in their entirety within the FOV); **b** FOV-B bed position includes AA and kidneys in their entirety

All PET/CT scans were performed on the same scanner (Siemens Biograph mCT; 64 slice-4R) with a 22-cm axial FOV. On each day of examination, the PET/CT scanner was quality checked and calibrated according to system-required procedures. A peripheral venous catheter (Venflon) was placed in a cubital vein for ^82^Rb injection. Subjects rested in a sitting position for approximately 30 min before voiding. They were then placed in a supine position in the PET/CT scanner with arms extended above the head and the generator infusion system connected to the Venflon. All subjects underwent two consecutive duplicate PET/CT scans. The duplicate scans were acquired in bed position A (FOV A), including the LVBP, the AA and as much of the kidneys as possible (Fig. 2a), and bed position B (FOV B), including the AA and the kidneys in their entirety (Fig. 2b). Computer-generated randomisation determined the acquisition sequence for the two FOVs for each participant. In each bed position, an initial planar scout image was acquired to determine positioning of the scanner over the required FOV. Following positioning, a low-dose CT scan was performed immediately followed by a bolus injection of 555 MBq ^82^Rb and a dynamic PET-scan in list-mode for 8 min synchronised with the start of injection [21]. Sequentially, and 10 min after the first PET scan was initiated, a second dose of ^82^Rb was administered, and a duplicate PET scan performed in list-mode for 8 min. The bed position was then shifted, and the procedure repeated for the second FOV.

Low-dose CT scans (25 mAs, 100 kV) were performed for attenuation correction purposes only. PET data were acquired in dynamic list-mode, which was re-binned using 32 frames (20×6 s, 5×12 s, 4×30 s, and 3×60 s) and iteratively reconstructed (21 subsets, 2 iterations) using Siemens TrueX and time-of-flight reconstruction in a matrix of 128×128 (voxel size 6.4 × 6.4 × 3.0 mm^3^) and post-filtered with a 5.0-mm Gaussian filter to produce attenuation and decay-corrected dynamic sequences. We found it unnecessary to correct for motion of the kidneys.

The effective radiation dose associated with the study was < 4 millisieverts (mSv): each low-dose CT scan contributed 0.4 mSv and each 555 MBq bolus injection of ^82^Rb contributed with 1.26 μSv/MBq [20].

### Analysis of ^82^Rb PET/CT studies

**Fig. 3:**
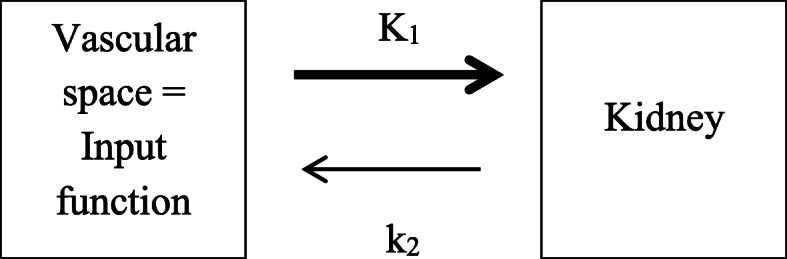
One-tissue compartment model used for estimation of RBF. K_1_ is the rate constant for ^82^Rb uptake in the kidneys from the vascular space, whereas k_2_ is the rate constant for release of ^82^Rb back into the blood. No discernible tracer activity was observed via urinary excretion [21]

A 1-tissue compartment model was used for flow estimation [21], as illustrated in Fig. 3. The K_1_ parameter represents the renal clearance of ^82^Rb, where K_1_ (ml/min/cm^3^) is equal to the product of the blood flow component carrying the ^82^Rb (erythrocyte and/or plasma) and its extraction fraction (EF) in the kidneys. Due to ^82^Rb having a high first pass extraction (~ 90%) [19], its uptake rate K_1_ will be closely related to, and hence can be used as, an estimate of flow [27]. Compartmental modelling was performed using the PMOD software (PMOD Technologies Ltd., Zurich, Switzerland, version 4.01).

As ^82^Rb is a small molecule, it will be distributed in the water phase in plasma and as such is freely diffusible in the interstitium and constitutes the extravascular background. This intercellular distribution is included in the input function, which is modelled using a 3-exponential model in PMOD with the background activity corrected using the β-correction procedure.

### Determination of recovery coefficients

Necessary correction of PET-data for technical measurement bias relating to both scanner calibration (conversion of image counts to activity concentration) and recovery coefficients (deriving from partial-volume (PVE) and spill-over effects) was applied based on phantom-measured recovery coefficients (β) accounting for the combination of all technical bias contributions relevant for a given VOI definition. Organ-specific β values (VOI size and relative placement with respect to delimitating structural boundary dependence) were applied to count data obtained by VOIs placed in the myocardial left ventricle, the abdominal aorta and the kidneys.

For simulation of the LV geometry, the NEMA-IQ phantom was used filled with a background to hotspot ratio 1:10, known ^82^Rb activity concentrations and the same reconstruction parameters as for the study; this determined β to be 0.71 for the LV. Similarly, a homogeneous home-made phantom simulating the AA/background geometry determined β to be 0.612 for the aorta. Additionally, a recovery coefficient β = 0.643—measured in a large, homogeneous phantom volume—was applied to the kidney-TAC data. In this case, due to large kidney volumes and careful placement of applied VOIs at a distance from the kidney boundary walls, effects from PVE and spill-over are negligible and do not need correction. However, as the Siemens system software did not itself correct for ^82^Rb count efficiency, manual correction for this in the kidney data was essential to ensure that the measured activity concentrations in the different organ VOIs remain relatively correct to each other, when corrected for all necessary scanner and reconstruction effects. Additional information regarding phantom determination of the organ-specific β-values is described in Additional file 1.

### Image-derived input functions and time-activity curves

**Fig. 4:**
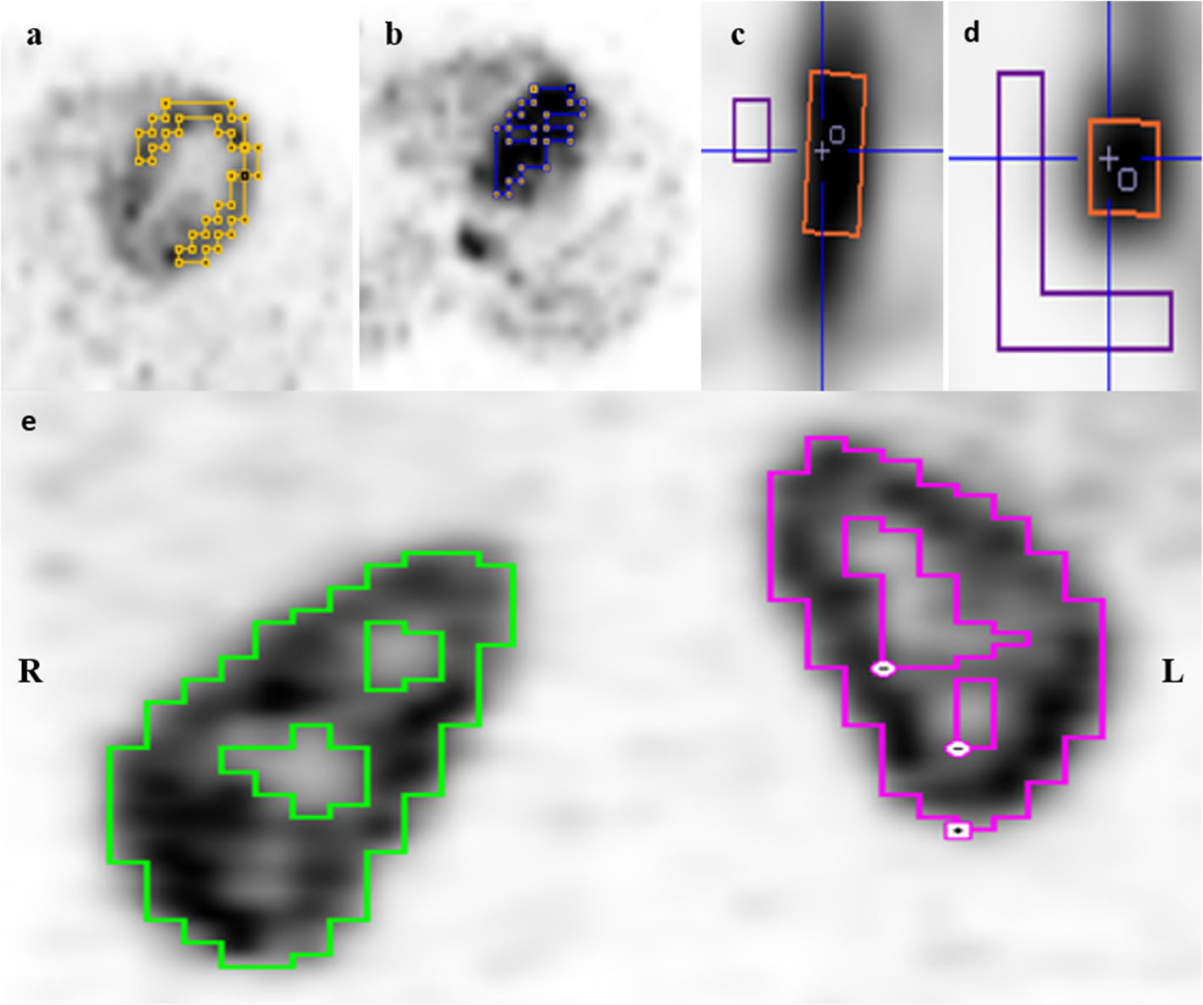
VOIs were drawn in **a** myocardium, **b** left ventricular blood pool, **c** and **d** abdominal aorta and aortic background (orange—aorta; purple—background) and **e** contouring the kidneys (green—right; cerise—left)

Time-activity curves (TACs) were obtained by defining relevant VOIs in the various anatomical regions of interest (Fig. 4), with the LVBP and AA defining IFs for the kinetic modelling. All TACs were obtained as the mean activity concentrations measured in the VOIs. The LVBP was defined in FOV-A using a limiting box and the hot-contour tool with a typical cutoff 45–60% of the maximum limiting box activity. Ensuring avoidance of surrounding activity in the right and left ventricular luminae, a background VOI was manually placed centrally in the left ventricular wall and defined on at least 10 contiguous slices. PVE and spill-over activity from the left ventricular wall was corrected adopting the method described by Katoh et al. [28]:

1 $$ {R}_A(t)=\beta \cdot {C}_A(t)+\left(1-\beta \right)\cdot \rho \cdot {C}_{Bg}(t) $$

where C_A_(t) represents the corrected LVBP activity, R_A_(t) is the measured LVBP activity, C_Bg_(t) the measured myocardial activity, *ρ* the partition coefficient of water in the myocardium (0.91) and β the recovery coefficient required to correct measured image activity concentration values to the correct activity present in the LVBP.

The AA-VOI was defined in both FOVs using a box (10×10×30 mm^3^) placed in the lumen of the abdominal aorta cranially to the departure of the renal arteries. An aortic background VOI was defined within a limiting box around the AA-VOI by applying a cold-contour with typical cutoff of 4–5% of the maximum activity and excluding all structures not representing background activity.

Based on the formulation for PVE and spill-over correction for the LVBP of [28], the measured AA activity concentration can be similarly corrected for possible background and PVE contributions using:

2 $$ {R}_A(t)=\beta \cdot {C}_A(t)+\left(1-\beta \right)\cdot {C}_{Bg}(t) $$

where C_A_(t) represents the corrected AA activity, R_A_(t) is the measured AA activity, C_Bg_(t) is the measured aortic background activity and β is the necessary recovery coefficient related to the AA geometry and analysis VOI placement.

Tissue TACs for both kidneys were obtained using hot contouring in both FOVs as described previously and K_1_ values for each kidney obtained for both LVBP and AA IFs using the 1-tissue compartment model. A blood volume fraction of 10%, applied as a fixed parameter in the PMOD kinetic modelling, was used to account for activity from the fractional blood volume within the VOIs contouring the kidneys [29].

Kinetic analysis was performed independently by two observers: a medical resident (observer 1) and an experienced nuclear medical physician (observer 2).

### Total renal clearance

Total renal clearance can be estimated using the measured ^82^Rb clearance (K_1_) and the total analysed kidney volume (V_Total_) as determined by the renal contour volumes described above:

3 $$ ERPF={K}_1\cdot {V}_{Total} $$

Clearance results are normalised to body surface area (BSA) using the Dubois formula:

4 $$ BSA=0.007184\cdot {height}^{0.725}\cdot {weight}^{0.425} $$

where quantity units are given in BSA [m^2^], height [cm] and weight [kg].

### Statistical analysis

Statistical tests were performed using SPSS Statistics ver. 20 (IBM Corp., Armonk, NY, USA). For each subject, the result for K_1_ was defined as the mean value of the two independent K_1_ values determined for each FOV for both input functions.

Values are presented as mean ± SD for all completing subjects. Paired sample t-testing was used for comparison of K_1_ values obtained using LVBP and AA IFs, where *p* < 0.05 was considered statistically significant.

Intra-assay coefficients of variation were calculated for each kidney based on the duplicate K_1_ determinations in each FOV. Inter-observer variability was assessed using the intra-class correlation coefficient (ICC) with 95% confidence interval (CI) [30].

## Results

### Demographics

**Fig. 5:**
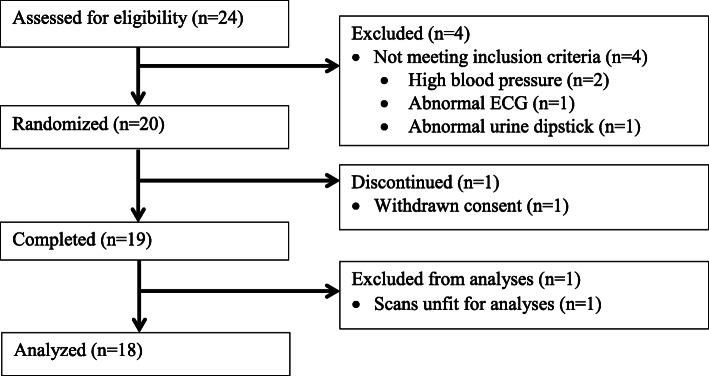
Participant flow in the study

The participation flow chart for the study is depicted in Fig. 5. Eighteen healthy subjects completed the study and had scans accepted for analysis. Clinical and biochemical characteristics are shown in Table 1.

**Table 1:** Clinical and biochemical characteristics (*n* = 18)

Age (years)	21 ± 4
Gender (women/men)	7/11
BMI (kg/m^2^)	24.1 ± 2.5
Office SBP (mmHg)	127 ± 9
Office DBP (mmHg)	73 ± 10
Heart rate (beats/min)	71 ± 11
P-alanine aminotransferase (U/L)	26 ± 11
P-sodium (mmol/L)	140 ± 2
P-potassium (mmol/L)	3.7 ± 0.2
P-albumin (g/L)	43 ± 3
P-creatinine (μmol/L)	73 ± 13
eGFR_MDRD_ (mL/min/1.73m^2^)	118 ± 11
B-haemoglobin (mmol/L)	9.0 ± 0.4
B-leucocytes (× 10^9^/L)	7.1 ± 1.9
B-thrombocytes (× 10^9^/L)	275 ± 48
B-haematocrit	0.42 ± 0.02

Data are presented as mean ± SD

*BMI*, body mass index; *SBP*, systolic blood pressure; *DBT*, diastolic blood pressure; *eGFR*_*MDRD*_, estimated glomerular filtration rate calculated using the Modification of diet in renal Disease Study equation; *EVF*, erythrocyte volume fraction

### Input curves

**Fig. 6:**
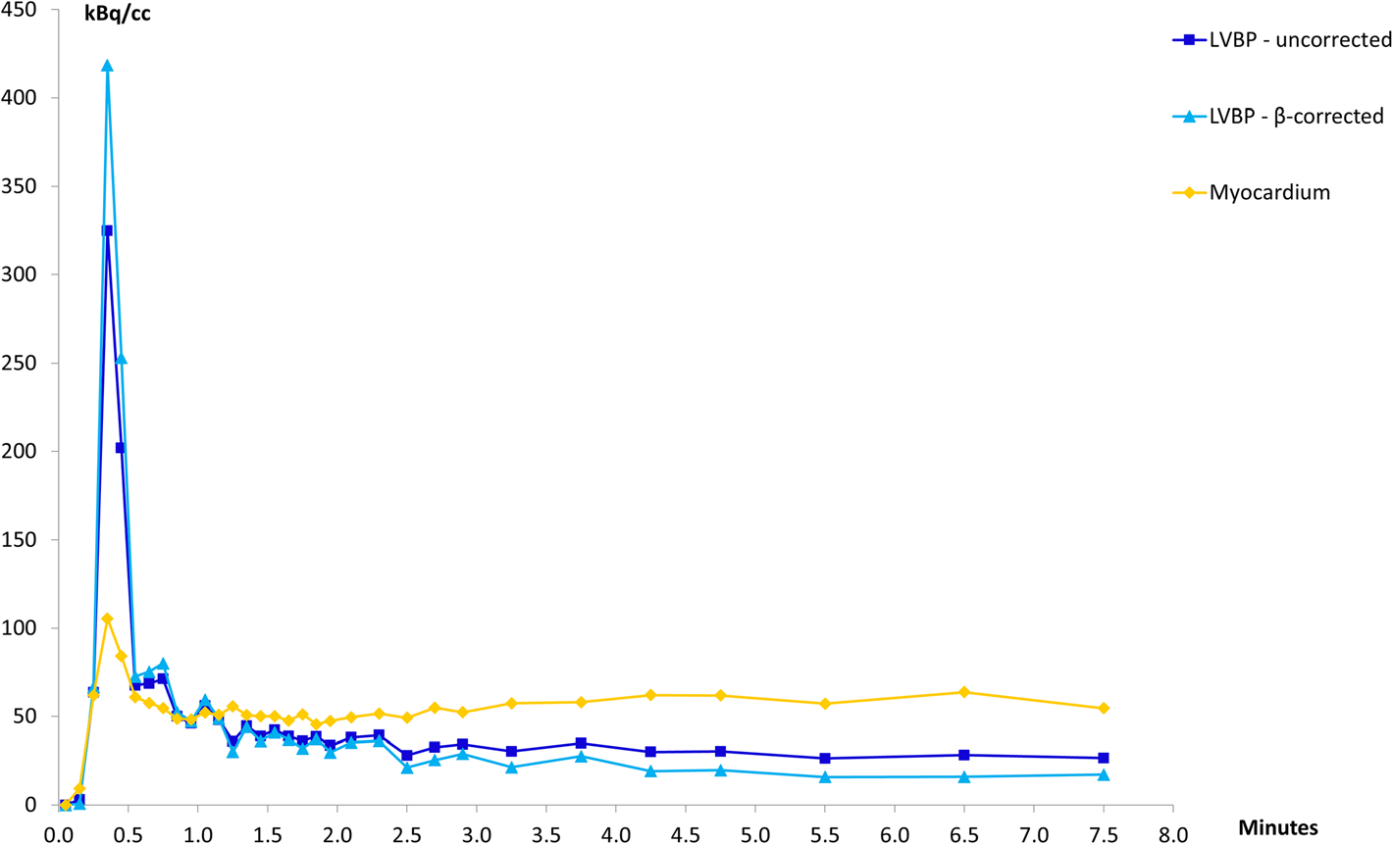
Representative time activity curves from the left ventricular blood pool and the myocardium from one of the study subjects

Figure 6 illustrates typical TACs generated from VOIs in the LVBP and the myocardium, as well as β-corrected activity in LVBP. For all curves, the activity peaks rapidly. However, whereas for LVBP the flow-peak is followed by a continuous decline, the myocardial activity plateaus around 1.0 min post-injection (p.i.), after the decline of the initial flow peak.

**Fig. 7:**
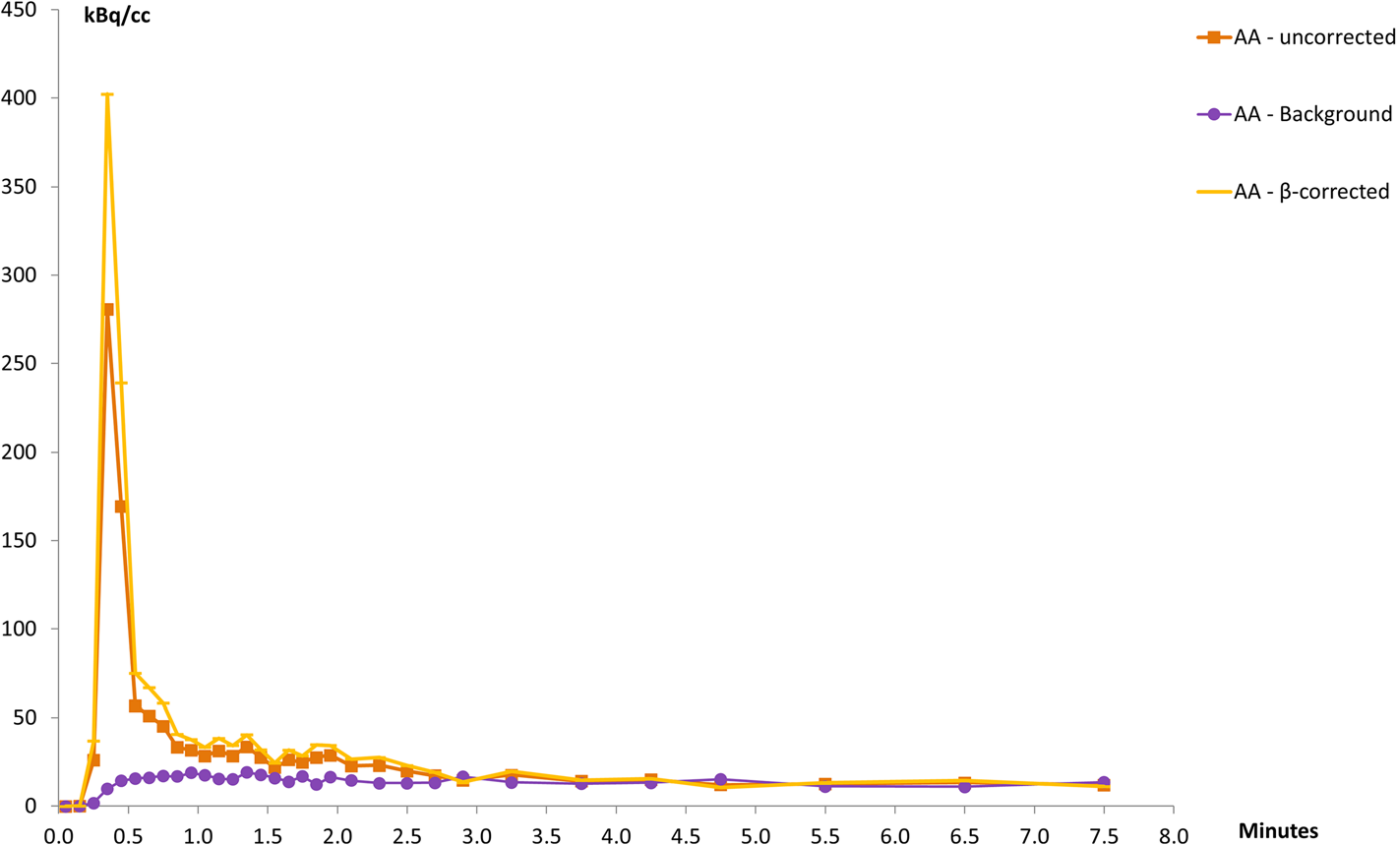
Representative time activity curves from the abdominal aorta and the aortic background from one of the study subjects

Similarly, Fig. 7 illustrates typical TACs generated from VOIs placed in the AA and the aortic background as well as the β-corrected AA activity. As for LVBP, AA activities rapidly reach their maximum peak followed by rapid declines, while the aortic background activity rises slowly until reaching a plateau between 0.5 and 3.0 min p.i. followed by a slow decline.

**Fig. 8:**
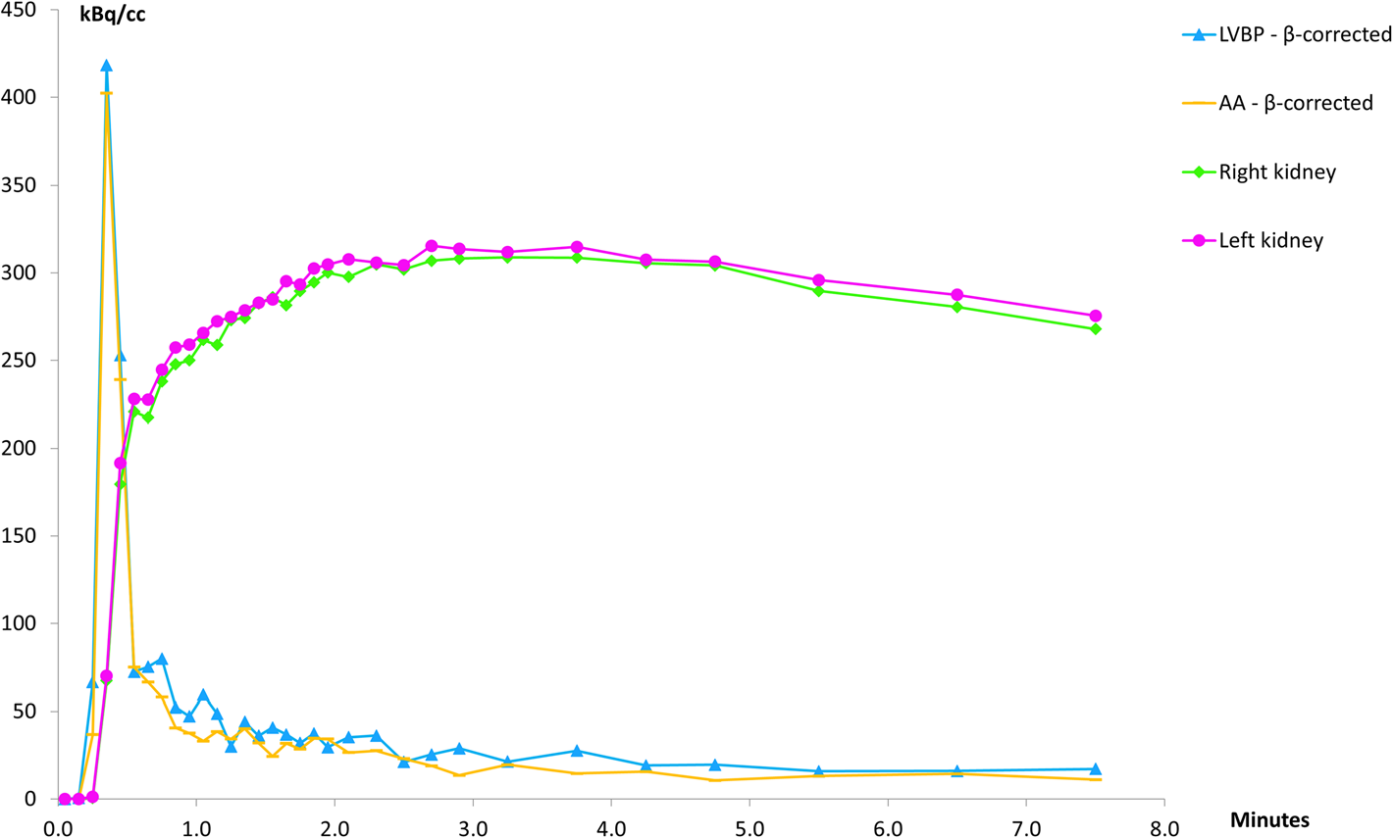
Typical time activity curves from the left ventricular blood pool, abdominal aorta and the kidneys from one of the study subjects. The observed presence of low activity in the kidneys before the activity in the blood pool has peaked is due to the automatic injection system for delivery of ^82^Rb providing an infusion of the radioisotope over a 20–40 s period, which does not constitute a true bolus injection

Figure 8 shows a typical example of the relative ^82^Rb activity concentrations (corrected TAC data) between the organ VOIs of the LVBP, AA and kidneys. The injected bolus peaks for the LVBP and AA are very similar, reaching nearly the same maximum peak values in the same time-bin after injection, whereas the kidney uptake rises more slowly until reaching a plateau between 1.5 and 4.5 min p.i. followed by a slow decline.

### Renal clearance—measurement of K_1_

**Fig. 9:**
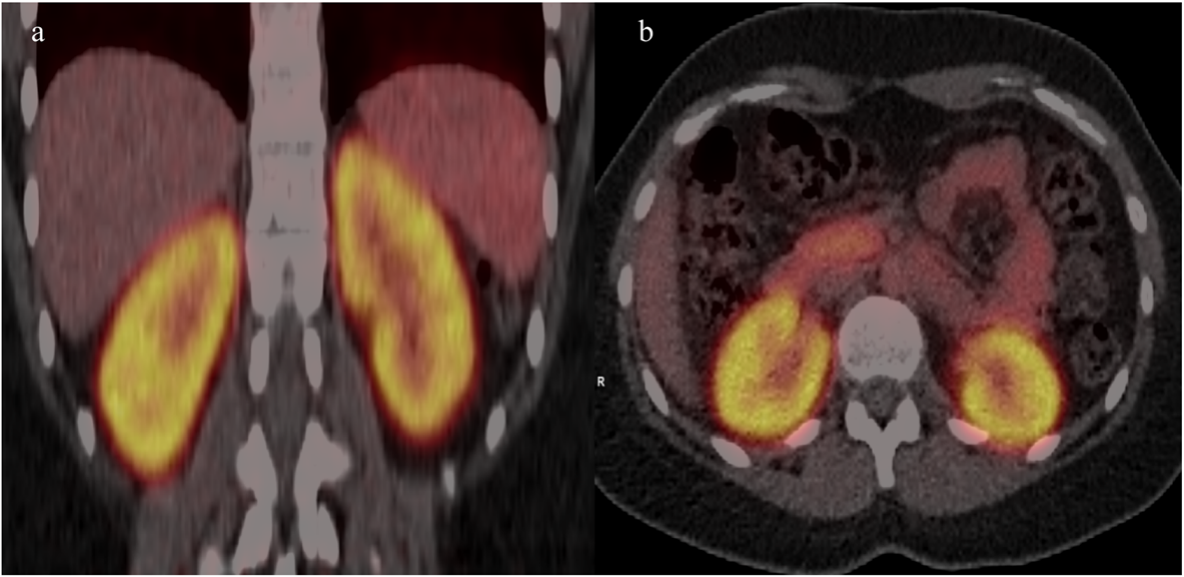
Typical example of **a** coronal and **b** transaxial PET/CT images of kidneys during maximal ^82^Rb uptake from one of the study subjects. Shown example is for FOV-B

High renal uptake of ^82^Rb was demonstrated with no discernible urinary activity (Fig. 9).

The caudal part of the kidneys was outside FOV-A in 5 out of the 18 completing subjects. Measurements in FOV-B showed that K_1_ for the excluded caudal sections did not differ from the global K_1_ for the kidneys.

Table 2 presents the mean K_1_ results for all IFs applied in the analysis. K_1_ values using the AA IF were not significantly different from those using LVBP. No significant difference was observed between the left and right kidneys.

**Table 2:** Mean K_1_ values for the investigated input functions

	LVBP	AA (FOV-A)	AA (FOV-B)
Right kidney	2.75 ± 0.42	2.86 ± 0.48*	2.82 ± 0.45^^^
Left kidney	2.71 ± 0.42	2.82 ± 0.45*	2.79 ± 0.42^^^

Data are presented as means ± SD. K_1_ units are ml/min/cm^3^

Paired t-test *NS vs. K1 values derived from LVBP

^NS vs. K1 values obtained from AA (FOVA)

*LVBP*, left ventricular blood pool; *AA*, abdominal aorta; *FOV-A*, field of view A; *FOV-B*, field of view B; *NS*, non-significant

Intra-assay coefficients of variation for the duplicate VOIs for each FOV were calculated for K_1_ derived from LVBP and AA IFs. As illustrated in Table 3, the intra-assay coefficients of variation were similar (~ 5%) for both IFs, with those for AA being slightly lower than those for LVBP IFs.

**Table 3:** Intra-assay coefficients of variation

	LVBP	AA (FOV-A)	AA (FOV-B)
Right kidney	5.6	4.4	4.4
Left kidney	5.7	4.3	4.4

Data are presented as percentages

*LVBP*, left ventricular blood pool; *AA*, abdominal aorta; *FOV-A*, field of view A; *FOV-B*, field of view B

Table 4 shows inter-observer variability when using LVBP and AA IFs for the two FOVs. Using LVBP, ICC was indicative of good to excellent reliability for both kidneys. For AA, ICC was suggestive of excellent reliability for both kidneys in FOV-A and FOV-B.

**Table 4:** Inter-observer variability

	LVBP	AA (FOV-A)	AA (FOV-B)
Right kidney	0.874 (0.696; 0.951)	0.971 (0.925; 0.989)	0.969 (0.920; 0.988)
Left kidney	0.880 (0.708; 0.953)	0.972 (0.926; 0.989)	0.965 (0.909; 0.987)

Data are presented as ICC estimates with 95% confidence intervals in parentheses

*LVBP*, left ventricular blood pool; *AA*, abdominal aorta; *FOV-A*, field of view A; *FOV-B*, field of view B

Clearance values were calculated using equations 3 and 4 and are presented in Table 5.

**Table 5:** Estimation of total renal clearance based on ^82^Rb clearance values (K_1_) using AA activity in FOV-B

Average K_1_ (ml/min/cm^3^)	Total volume analysed (cm^3^)	Total clearance (ml/min)	Body surface area (m^2^)	Total clearance normalised (ml/min/1.73 m^2^)
2.80 ± 0.43	296 ± 30	825 ± 122	1.87 ± 0.16	766 ± 114

Data are presented as means ± SD. Total clearance is calculated as the product of K_1_ and the total volume of renal VOIs

## Discussion

This study confirms that estimation of renal perfusion based on ^82^Rb PET/CT using AA as the IF in a 1-tissue compartment model is feasible, as previously indicated by Tahari et al. [21]. Additionally, our results support the use of the AA-VOI in a single FOV as an alternative IF to the LVBP; the low intra-assay coefficients of variation are acceptable with excellent inter-observer reliability, thus allowing renal clearance of ^82^Rb to be determined using a single FOV assessment of the kidneys in their entirety. To our knowledge, this is the first study to assess method precision and determine intra-assay variation and inter-observer variability for clearance estimates with ^82^Rb PET/CT.

### ^82^Rb as renal perfusion tracer

There are many advantages to using ^82^Rb PET/CT for measurement of renal perfusion: it is non-invasive and does not require blood sampling or urine collection, making the procedure less burdensome for patients; it allows for single kidney perfusion analysis and is readily available from ^82^Sr/^82^Rb generators which are already in situ at sites routinely using ^82^Rb for assessment of myocardial blood flow, thus making it cost effective. In comparison, the “ideal tracer”—^15^O-water—can be utilised only in centres with on-site cyclotron access [31]. The combination of a short ^82^Rb half-life of 75 s and short acquisition time allows for repeated scans of the same subject within a short timeframe, presenting unique opportunities to examine acute effects of differing drugs on renal perfusion. For example, ^82^Rb PET/CT may be especially suitable for use in cross-over studies exploring interventional effects.

No absolute contraindications exist to the use of ^82^Rb; thus, patients suffering from all stages of AKI and CKD can undergo the examination without risk of deterioration of renal function.

Since renal ^82^Rb accumulation exceeds myocardial ^82^Rb accumulation, half the tracer dose of cardiac studies is sufficient to perform good quality renal imaging, resulting in a low effective radiation dose (~1 mSv) for a single scan of the kidneys in their entirety, including the AA for use as IF. Additionally, for modern digital scanners with high sensitivities, even lower tracer doses may be sufficient to perform the examination.

### Input functions and necessary data correction

Pharmacokinetic modelling requires an IF, where sampling of peripheral arterial blood to produce an arterial TAC is the gold standard method for obtaining an accurate estimation. However, the short half-life of ^82^Rb necessitates an alternative to the arterial sampling derived input curve. This can be achieved using image-derived input curves based on, e.g., PET/CT scanning, where LVBP- and AA-TACs are examples of IDIFs. Accurate quantitative IDIF estimation is dependent on many parameters, relating to both the individual PET/CT scanner and reconstruction parameters used for imaging, the geometry, size and placement of analysis VOIs with respect to structural organs-of-interest boundaries, the ratio of neighbouring activity concentrations, as well as requiring calibration of the ^82^Rb-tracer injector system and imaging scanner with associated dose calibrators, the 3 primary sources of quantitation error being scanner count efficiency, PVE and spill-over. As a minimum, an understanding of what corrections are, and are not, automatically included in an individual scanner-systems software, is necessary to verify correct method implementation and data analysis locally. If one can ensure the relative contributions from PVE and spill-over are negligible in all VOIs used to obtain organ-specific TAC data, the assumption that any global scanner-specific error will cancel out in the kinetic modelling should be adequate and allow for evaluation of activity concentrations without need for cross-calibration of all systems. However, if it is not possible to ensure negligible PVE and spill-over effects in one or more of the VOIs, then, the relative activity concentrations defining the TACs will not be correct with respect to each other and will result in an erred kinetic analysis of renal flow. One method to reduce PVE is to define VOIs as 1-cm^3^ volume spheres centred on the highest activity voxel in the organ of interest and measure peak-activity concentrations. However, placement of smaller VOIs is variable and observer dependent, especially in highly inhomogeneous (biological) activity distributions. As such, it can be advantageous to use mean values to define the activity measurements. Additionally, if the maximum voxel count lies in proximity to an organ boundary, PVE and spill-over will not be reduced and will still have to be accounted for in the data analysis.

We obtained uncorrected IDIFs from TACs based on VOIs placed in both LVBP and AA in the dynamic PET images. Use of IDIFs based on large-size vascular structures, combined with the high resolution of modern PET scanners, reduces PVE in activity measurement [32, 33]. Additional investigation of measured activity accuracy as a function of distance from structural boundaries (specific to our scanner and reconstruction method), using a phantom containing known ^82^Rb activity concentrations in geometrical structures simulating the volumes, shapes and sizes of the LVBP and AA (Additional file 1), showed that to ensure negligible PVE and spill-over effects when defining a VOI, its placement needs to be a minimum of 15 mm from organ boundaries, i.e., a minimum of 3–5 voxel distances, dependent on the choice of imaging matrix. In the smaller AA structure, even though there is very little background to give unwanted spill-in, this criterion was not met, indicating that PVE is present and requires correction. On the other hand, the large LVBP volume indicates that PVE is reduced. However, due to significant uptake of ^82^Rb activity in the left ventricular wall and the use of hot-contouring producing VOIs with, at most, 1–2 voxel distances from the myocardium, the LVBP also required correction for PVE and spill-over. Both corrections were performed based on the method of Katoh et al. [28] using Eqs. 1 and 2, with the necessary correction factors experimentally determined from phantom measurements. Additionally, a global calibration of our scanner’s count efficiency in a large (> 100cm^3^) homogeneous volume was made, to provide kidney VOI data correction. Here, PVE and spill-over are negligible, but counting efficiency was not automatically corrected by the Siemens scanner software. Based on these arguments, three differing values for “organ-specific” β values were required to ensure the correct relative relationships between the corrected organ-TAC data; one cannot assume that a single, global scanner and reconstruction-dependent correction factor will cancel out in subsequent kinetic analysis, unless the employed VOI definition protocol ensures independence from PVE and spill-over in all organs.

Comparison of our IFs with those of the first (and to date only published) human renal ^82^Rb PET/CT study by Tahari et al. [21] show both similarities and differences. In both studies, the uncorrected activity in the AA is observed to be lower than LVBP activity. As there is no known metabolism of ^82^Rb in its passage through the aorta, it is assumed that the activity concentrations in the left ventricle and the aortic lumen are equal, and as such, observed measurement differences will be caused by any scanner and image reconstruction quantification inaccuracies, as discussed above. Tahari et al. [21] assessed the effect to arise from PVE and performed the correction using a simple scaling of their measured AA activity to match the observed maximum LVBP activity. It is unclear whether LVBP activity in [21] was corrected for PVE and spill-over. Our more systematic approach, in which calibrated phantom measurements determined the recovery coefficient β necessary to correct VOI specific activity measurements for a given organ geometry, gave β values of 0.71, 0.612 and 0.643 respectively for LV, AA and kidney TAC corrections, where the numerical value for β does not differentiate between the relative contributions from PVE, spill-over or count efficiency, but provides a “global” factor accounting for all contributions. Application of these organ-specific β values increased the measured peak values for both LVBP and AA TACs (Figs. 6 and 7) resulting in the corrected-AA IFs being scaled to match the corrected LVBP IFs (Fig. 8), supporting the assumption that activity concentrations in the left ventricle and aortic lumen are equal. This agrees with the IFs shown in Tahari’s study. The main difference is that our K_1_ values obtained from AA IFs differ from their AA K_1_-derived values, due to our correction of kidney-TACs for system counting efficiency.

Using the β-corrected TAC data, we find for both AA and LVBP, the intra-assay coefficients of variation are acceptably low, indicating that ^82^Rb PET/CT is a precise method for evaluation of K_1,_ hence allowing for determination of changes in K_1_. Additionally, the inter-observer variability assessment supports the use of AA as IF as a robust image-derived method for determining renal perfusion, with excellent reliability demonstrated for both kidneys using AA, compared to good to excellent reliability using LVBP.

### Renal clearance—measurement of K_1_ and interpretation of clearance values

High renal ^82^Rb uptake and accumulation were confirmed. To minimise errors in uptake estimation caused by regional differences, it is important to measure uptake in the entire kidney. In our study, 13 out of 18 completing subjects (72%) showed both LVBP and the entire kidneys in FOV-A, such that 5 analyses were performed on truncated kidneys. In the article by Tahari et al. [21], only 3 out of 8 subjects (38%) had the LVBP and kidneys in the same FOV (corresponding to FOV-A), and 5 out of 8 subjects had the LVBP and kidneys in separate acquisitions. As a global quality control, we found no significant difference between K_1_ values derived from AA activity curves in FOV-A and those in FOV-B, supporting the assumption that in the studied population with healthy, lesion-free kidneys, quantitation obtained from truncated images of the kidney tissue is representative of values which would be obtained from imaging the kidneys in their entirety. This may not, however, be universally true. Since blood flow differs between the renal cortex and outer and inner medulla, the extent of differential renal tissue included in the analysis VOI may affect values of measured blood flow.

The poor quality of the low-dose CT, used for AC-correction only, did not allow discrimination of the cortex and medulla in our renal VOIs, and even the relatively good quality images and high renal uptake observed in ^82^Rb PET/CT imaging could not ensure a reliable discrimination between diverse flow regions. This differential flow measurement is potentially possible using CT contrast enhancement or even PET/MR from which to define the kidney VOIs, but was not available for the present studies.

The conversion of K_1_ values (ml/min/cm^3^) to total clearance values for both kidneys (ml/min) can be approximated by multiplying the K_1_ values with the total volumes of the renal VOIs assuming 1 cm^3^ to be equivalent to 1 g of tissue. These clearance values are summarised in Table 5.

The calculation of total clearance is dependent on the measurement of the K_1_ values, which are observed to be quite robust and are only to a limited extent dependent on the size of the VOIs used in their determination. However, the delineated volumes of the renal VOIs provide only a rough approximation to the actual anatomic volumes examined. This is due to the less-than-optimal resolution of the PET-scanner, which for this study used a matrix with voxel size 6.3 × 6.3 × 3 mm corresponding to a voxel volume 0.12 cm^3^ and can lead to errors in determination of the volume analysed: it is likely that our renal VOI volumes are somewhat overestimated. This is based on the observation that the average total volume used for analysis was 296 ± 30 ml, corresponding reasonably to the total anatomical VOI for both kidneys being 300 ml. This corresponds to the reported value for “total parenchymal three-dimensional volume” of 302 ml (range 215–499 ml) as determined by CT-volumetry in a recent study by Gardan et al. [34]. As this volume corresponds to the entire kidney volume, including low flow regions contributing little to the total clearance, then the actual “renal volume” responsible for high flow rates must be smaller. In the same CT-volumetry study, the “cortical renal volume” was measured to be 189 ml (range 126–308). Use of this volume would render total clearance values of 527 ml/min and a BSA normalised total clearance of 489 ml/min, but would probably provide a slightly underestimated value for the total clearance. This illustrates that accurate determination of total clearance values based on PET/CT methodology requires accurate knowledge of the renal volume of relevance. Thus, to obtain a reliable estimation of total clearance using ^82^Rb, future studies will need to have a better, and preferably standardised, definition of the regions responsible for the flow values in question, which will then allow for reliable comparison to existing reference methods as, at least for ERPF determination, these are independent of this variable.

or this study, our measured total clearance values are observed to be low when compared to previously published mean values for RBF: ~1100–1500 ml/min [35, 36]. However, they are quite similar, if somewhat at the high end, to previously published mean values for ERPF with values 345–700 ml/min/1.73 m^2^ [35, 37, 38]. This suggests ^82^Rb PET/CT may actually be estimating ERPF and not RBF as is the current understanding.

Whether we measure estimated RBF or ERPF with ^82^Rb depends on the distribution of the tracer between plasma and erythrocytes in whole blood. Early studies on potassium permeability of the erythrocyte membrane showed a very slow exchange of radioactive potassium and rubidium between plasma and erythrocytes amounting to 1.8–2.1% per hour and even less over 8 min of study [25, 26]. Hence, most ^82^Rb is present in the plasma during renal uptake studies, implying that the measured renal uptake values most likely represent estimated RPF after correction for extraction, if EF is close to unity.

Assuming our data represents RPF, estimated RBF can be calculated by correcting with the haematocrit value which is easily measured. For canines, EF is estimated to be 0.89 (0.80–0.95) [19], but to our knowledge, remains to be determined in humans due to difficulty in calibrating and measuring blood activity for ^82^Rb. However, if we assume the extraction values to be similar for humans, after correction for the haematocrit value (in the present population 42 ± 0.02), we find an average total estimated RBF value normalised to BSA of 1494 ± 221 ml/min/1.73 m^2^, which lies at the upper end of the expected general range for RBF in healthy subjects. Since our study population consisted of a highly homogeneous group of young, healthy subjects, our estimation of a high ERPF and consequently a high RBF is to be expected; our results being consistent with 2 previous studies of similar population groups, published in 1959 and 1960 respectively [35, 36], where mean RBF values in the range 1100–1500 ml/min, were calculated based on measurement of PAH-clearance and using conversion for extraction fraction and haematocrit. Specifically, our mean and range of estimated RBF values are fully consistent with the published range of individual RBF values (1150–2350 ml/min) in the study by Brodwall et al. [36].

### Study strengths and limitations

The major strengths of this study are a combination of the randomised cross-over design, the standardisation of pre-scan conditions (fluid intake, exercise level, duration of fasting period), and the consecutive acquisition of the four ^82^Rb PET/CT scans over a short 45-min period, enabling optimal evaluation of intra-assay coefficients of variation and hereby precision. Additionally, the use of measured recovery coefficients provides reliable numerical correction of the IFs which are specific to our PET/CT scanner, imaging reconstruction method and choice of VOI definitions.

The homogeneous study population consisted of healthy adults, providing estimated RBF measurements uninfluenced by age and medical therapy. However, this is also a potential bias as it is not certain that results from this study can be directly applied to a population of elderly subjects, nor to subjects suffering from hypertension or renal disease; additional feasibility studies may be needed for these populations. Additionally, before ^82^Rb PET/CT can be implemented for clinical estimated RBF determination, further evaluation is required of day-to-day variation as well as the quantitative accuracy of the method.

A technical limitation is the use of the automatic injection system used to provide the bolus administration of activity; in practice, a short infusion of ^82^Rb is administered, which depending on the age of the ^82^Sr/^82^Rb generator can have a duration between 20 and 40 s, and as such does not represent a true bolus injection which should ideally be administered within 10 s. For this reason, low activity may be present in the kidneys even before the activity in the blood pool has peaked, as seen in Fig. 8.

Another limitation, when estimating RBF from the measured K_1_ values, is the assumption that the extraction fraction and blood volume fraction, as derived from animal experiments, are valid for calculation of RBF in humans from measurement of ERPF. Due to a general lack of published literature for ^82^Rb renal flow measurement, with early research based nearly exclusively on animal studies (1990s and earlier) and the first (and as far as we are aware, only) human investigation performed and published by Tahari et al. in 2014, no solid data is available for humans, such that animal-based values are the best, and only, data available to us.

Also, a significant limitation is that it does not provide comparison to a reference method; the accuracy of ^82^Rb PET/CT for RBF estimation cannot be evaluated. Based on the available literature during study design, the assumption was that the method provides a measurement of RBF for which an appropriate reference method would be in comparison with ^15^O-water studies [31]. However, in light of the results presented here, if we are in fact measuring plasma flow and not, as originally assumed RBF, then additional reference methods become available, such as PAH/OIH-clearance methods [35, 37, 38]. In fact, comparison of ^82^Rb measured K_1_ flow values, with a reference method for ERPF evaluation, could help answer the question regarding which quantity is actually being measured in ^82^Rb PET/CT studies. However, as discussed above, the accurate calculation of total clearance/flow rates is highly dependent on the volume analysed, and for future studies, a standardised segmentation of the regions analysed will be needed to provide accurate analysis and comparison with these reference methods.

## Conclusion

The results presented in this study, for a population of young, healthy subjects, support the use of an AA IDIF in the 1-tissue compartment model as an alternative to LVBP; it is sufficient to determine estimated total clearance/flow rates using a single FOV including AA and kidneys in their entirety using a single dynamic ^82^Rb PET/CT scan. Accurate quantification of the AA-derived IDIF requires PVE corrections, and eventual count efficiency calibration, relevant for the imaging scanner and reconstruction method employed. Use of AA gave rise to an acceptably low intra-assay coefficient of variation (~5%) and good to excellent inter-observer reliability.

Our data suggest that the actual flow values measured by ^82^Rb most likely represent ERPF rather than RBF which is essential for the correct interpretation of future perfusion studies using ^82^Rb. As the accurate calculation of total clearance/flow is dependent on the volume analysed, for future studies, we need a standardised definition of the renal VOI volumes analysed to allow proper comparison with existing reference methods for ERPF and RBF determination.

## Supplementary Information

**Additional file 1.** Appendix: 82Rb-phantom studies for determination of recovery-coefficients.

## Data Availability

The datasets and trial protocol (Danish) are available from the corresponding author on reasonable request.

## Abbreviations

AA: Abdominal aorta
AKI: Acute kidney injury
ALAT: Alanine aminotransferase
BMI: Body mass index
BSA: Body surface area
CI: Confidence interval
CKD: Chronic kidney disease
CT: Computed tomography
EF: Extraction fraction
ERPF: Effective renal plasma flow
FOV: Field of view
ICC: Intra-class correlation coefficient
IDIF: Image-derived input function
IF: Input function
LVBP: Left ventricular blood pool
^15^O water: Oxygen-15 labelled water
PET: Positron emission tomography
p.i.: Post-injection
PVE: Partial volume effect
^82^Rb: Rubidium-82
RBF: Renal blood flow
^82^Sr: Strontium-82
SD: Standard deviation
TAC: Time activity curve
VOI: Volume of interest

## Footnotes

1 Danish alcohol unit = 15 ml (12 g) pure alcohol.
