# Supplementary material for: Estimation of renal perfusion based on measurement of rubidium-82 clearance by PET/CT scanning in healthy subjects
Source: EJNMMI Phys. 2021 May 31;8:43. doi: 10.1186/s40658-021-00389-0 (PMC8167076; doi:10.1186/s40658-021-00389-0)
Supplement: Supplementary file 1 — Additional file 1. Appendix: 82Rb-phantom studies for determination of recovery-coefficients. [file 40658_2021_389_MOESM1_ESM.docx]

# Appendix: ^82^Rb-phantom studies for determination of recovery-coefficients

**Background:**

Phantom-studies were performed to evaluate the necessary correction factor(s) – β-recovery coefficients – required to account for scanner counting efficiency, effects of partial volume (PVE) and spill-in/spill-out (i.e., “spill-over”) in the measured activity concentrations from our acquired PET/CT study data. These results are specific to our Siemens Biograph mCT (4 ring/64 slice) PET/CT scanner (Department of Nuclear Medicine, Regional Hospital Gødstrup, Denmark).

**Methods:**

Known activity concentrations, with values resembling those observed in vivo, were measured in 3 different phantom-settings in 4 separate tests in a homemade phantom.

The phantom (Fig. 1) was constructed out of a water-filled 1 L measuring jug in which a water/activity filled 60 ml syringe was submerged to simulate acceptably realistic anatomical aorta/LV geometry and volumes, allowing for evaluation of (1) scanner count efficiency, (2) PVE & spill-out effects and (3) PVE & spill-in effects.

Challenges in accurate determination of the known phantom activity concentrations for the short-lived ^82^Rb isotope, were overcome using a Capintec 55TR dose calibrator which was time-synchronized to the PET-scanner's internal clock. Accurate activity measurement was achieved using a time-stamped (to the second hh:mm:ss) photo of the dose measurement allowing for accurate recording of the rapidly changing dose display. All dilution volumes and rest activities were obtained based on weighing syringes and liquid holders. Known activity concentrations were calculated and decay corrected to the PET-scan start time.

**Acquisition, reconstruction and data analysis:**

The same acquisition and reconstruction protocol was used in the phantom studies as for the article-study: Static-image reconstruction was performed by the PET-scanner system acquisition computer using Siemens True X iterative reconstruction (2 iterations / 21 subsets), 128 matrix, 5mm Gauss filter. Images were then transferred to PMOD for VOI drawing and quantification analysis.

Known concentrations are given as mean ± std for 5 repeated phantom measurements.

***1. Evaluation of (global) scanner correction for counting efficiency:***

Large homogenous VOI volumes (>100 ml) in a 500 ml beaker. Activity 0.99 ± 0.25 MBq/ml. The large volume minimizes effects of PVE and spill-over.

***2. Evaluation of recovery coefficient^[[1]](#footnote-1)^ (β) contribution from organ to surroundings:***

Homogenous activity in a 60 ml syringe with an inner diameter of 25 mm (dimensions resembling left ventricle or the diameter of the aorta). The Syringe was immersed in a water filled beaker with no “background activity” making it possible to measure effects of PVE and spill-out of luminal activity into the syringe wall and the surrounding water-filled background (Fig. 2). The average activity in the syringe was 0.74 ± 0.12 MBq/ml.

***3. Evaluation of recovery coefficient^1^ (β) contribution from surroundings to organ:***

Homogenous activity of 0.19 ± 0.06 MBq/ml in a water filled beaker in which a water filled, no activity, 60 ml syringe was immersed for measurement of PVE and spill-in of activity from “background” into the “lumen” of the syringe (Fig. 3).

***4. Investigation based on standard NEMA IQ-phantom setup for background to hotspot ratio 1:10:***

Studies with activity filled spheres (0.548 MBq/ml) in an activity filled background (0.057 MBq/ml) for calculation of β simulating the conditions in the body for activity in the left ventricle surrounded by activity in the myocardium (Fig. 4).
The recovery coefficient was calculated using the formula: β = (R_A_(t) - C_Bg_(t)) / (C_A_(t) – C_Bg_(t)), where R_A_(t) is measured activity, C_A_(t) is the true activity in lumen and C_Bg_(t) is the true activity in the background.

**Results and observations:**

In experimental series (1) we found that for our experimental acquisition and reconstruction parameters, the measured VOI activity concentration values for ^82^Rb were not automatically corrected under image reconstruction by the Siemens system.

Counting efficiency was calculated as the ratio between measured and true activity concentrations. On average we found a counting efficiency of 0.643 ± 0.014 for activity concentrations measured in large volumes (average = 100.2 ± 12.1 ml) using average known activity. This coefficient was used for correction of activity in the kidneys where VOI geometry and placement was optimized to eliminate effects from PVE and spill-over.

In experimental series (2) and (3) it is not possible to identify the individual effects of PVE and spill-over adjacent, and with increasing distance, from the holder boundaries, or in fact isolate counting efficiency in different VOIs: The “universal recovery coefficient” is a combination of all the factors influencing the measured activity compared to the true activity at a given position in the image geometry; it is dependent on the volume of the VOI and its distance from limiting borders such as the myocardium or the aortic wall.

Thus, for a cylindrical volume (diameter = 2 voxels of 6,3 x 6,3 mm) of ca. 4 ml in the centre of the “aorta”, β = 0.61 ± 0,03.

Study (4) using the NEMA IQ-phantom proved to be extremely difficult, being nearly impossible to fill both the spheres of the phantom and background volume within the short half-life of ^82^Rb with relevant activities. This problem is exacerbated by the time required to enable correct activity and volume measurements - the start activity concentration is extremely low at the PET-scan start time. The use of very large amounts of activity to overcome this is prohibited due to exposure from high radiation doses.

However, from the one successful study, we found β = 0.71.

**Fig. 1. Phantom used for determination of recovery coefficients (β)**


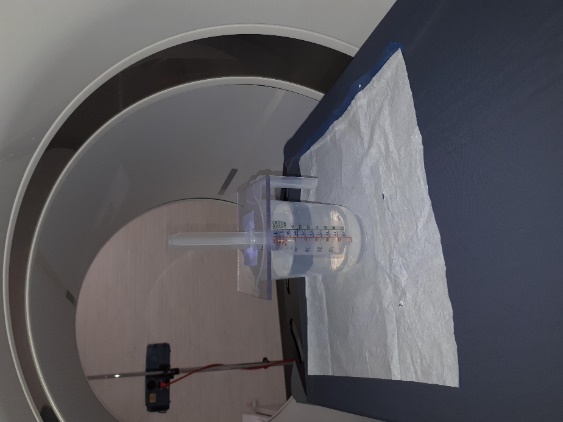


Figure 1. Home-made PVE-spill-over phantom to reproduce acceptably realistic anatomical aorta/LV geometry and volumes. The figure shows a measuring jug filled with ca. 700-750 ml water a 60 ml syringe with 50 ml water. The syringe is taped in place to prevent floating (movement artefacts) in the surrounding water.

**Fig. 2. PVE and Spill-Out of activity**


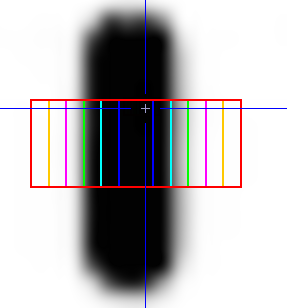


Fig. 2A Activity in syringe with no surrounding background contribution. Analysis VOIs are concentric cylinders positioned around the centre of the syringe.

Fig. 2B Extent of spill-out effects for ^82^Rb: Activity measured in concentric cylindrical VOIs around the centre of the syringe

**Fig. 3. PVE and Spill-In of activity**


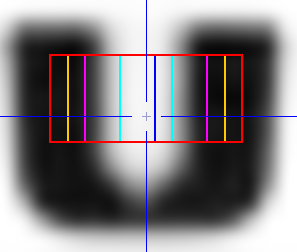


Fig. 3A. Background activity in beaker surrounding the adjacent "organ" syringe containing no activity. Analysis VOIs are concentric cylinders positioned around the centre of the syringe.

Fig. 3B. Extent of spill-in effects for ^82^Rb: Activity measured in concentric cylindrical VOIs around the centre of the syringe

**Fig. 4. IQ-Phantom**


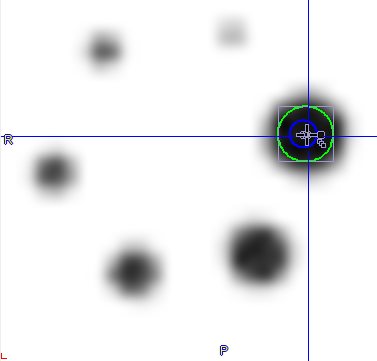


Fig. 4. The NEMA IQ-Phantom was filled with activity in spheres and in background in the ratio 10:1. Spherical VOIs with a diameter of 2 (12.6 mm) and 4 voxels (25.2 mm) were used for measurement of activity.

1. The total measured correction factor also includes the necessary contribution for ^82^Rb count efficiency, as this is not corrected automatically in the acquired list-mode data. I.e. recovery coefficient β = combined factor for correction of count efficiency, PVE and spill-over for the given geometry. [↑](#footnote-ref-1)
